# Embryonic Chicken Transplantation is a Promising Model for Studying the Invasive Behavior of Melanoma Cells

**DOI:** 10.3389/fonc.2015.00036

**Published:** 2015-02-16

**Authors:** Aparna Jayachandran, Sonja J. McKeown, Briannyn L. Woods, Prashanth Prithviraj, Jonathan Cebon

**Affiliations:** ^1^Cancer Immunobiology Laboratory, Ludwig Institute for Cancer Research, Melbourne-Austin Branch, Heidelberg, VIC, Australia; ^2^Department of Medicine, University of Melbourne, Melbourne, VIC, Australia; ^3^School of Cancer Medicine, La Trobe University, Melbourne, VIC, Australia; ^4^Department of Anatomy and Neuroscience, University of Melbourne, Melbourne, VIC, Australia

**Keywords:** embryonic chicken transplantation, melanoma, epithelial-to-mesenchymal transition, invasion, neural crest cells

## Abstract

Epithelial-to-mesenchymal transition is a hallmark event in the metastatic cascade conferring invasive ability to tumor cells. There are ongoing efforts to replicate the physiological events occurring during mobilization of tumor cells in model systems. However, few systems are able to capture these complex *in vivo* events. The embryonic chicken transplantation model has emerged as a useful system to assess melanoma cells including functions that are relevant to the metastatic process, namely invasion and plasticity. The chicken embryo represents an accessible and economical 3-dimensional *in vivo* model for investigating melanoma cell invasion as it exploits the ancestral relationship between melanoma and its precursor neural crest cells. We describe a methodology that enables the interrogation of melanoma cell motility within the developing avian embryo. This model involves the injection of melanoma cells into the neural tube of chicken embryos. Melanoma cells are labeled using fluorescent tracker dye, Vybrant DiO, then cultured as hanging drops for 24 h to aggregate the cells. Groups of approximately 700 cells are placed into the neural tube of chicken embryos prior to the onset of neural crest migration at the hindbrain level (embryonic day 1.5) or trunk level (embryonic day 2.5). Chick embryos are reincubated and analyzed after 48 h for the location of melanoma cells using fluorescent microscopy on whole mounts and cross-sections of the embryos. Using this system, we compared the *in vivo* invasive behavior of epithelial-like and mesenchymal-like melanoma cells. We report that the developing embryonic microenvironment confers motile abilities to both types of melanoma cells. Hence, the embryonic chicken transplantation model has the potential to become a valuable tool for *in vivo* melanoma invasion studies. Importantly, it may provide novel insights into and reveal previously unknown mediators of the metastatic steps of invasion and dissemination in melanoma.

## Introduction

Melanoma is a frequent malignant neoplasm, and metastasis of melanoma is the main cause of death in these patients (1–3). Metastasis is a complicated, multi-step process that is still poorly understood. Model systems have been developed to recapitulate cellular invasion, which is the early crucial step of the metastatic cascade (4, 5). Transwell invasion assays using the reconstituted Matrigel in Boyden chamber inserts have been utilized to study melanoma cell invasion *in vitro* (6, 7). However, the lack of *in vivo* microenvironmental factors may confound the results. Due to the transient and rare nature of the invasive process, there is a paucity of techniques for studying and visualizing motile melanoma cells *in vivo*.

Here, we describe a model using transplantation of melanoma cells into the neural tube of the embryonic chicken that is gaining traction for melanoma tumor invasion studies *in vivo*. This model was originally reported by Drews et al. for assessing melanoma cell behavior *in vivo* (8) and has been subsequently used by several other groups (9–13). It involves injecting melanoma cells into a microenvironment that is populated with neural crest cells that undergo an epithelial-to-mesenchymal transition (EMT) to exit from the neural tube and undergo extensive migration, to eventually populate a great diversity of areas in the embryo (14, 15). The developing chicken has been used extensively as a model to study developmental EMT and neural crest biology since the neural crest cells give rise to a wide variety of cell types including melanocytes, peripheral neurons and glia, secretory cells of the medulla, and bone and cartilage cells in the head (16–19).

Recently, this model has been adapted for studying cancer cell invasion since cancer cells use molecular programs, which are comparable to those utilized by migrating neural crest cells in the embryo (16, 20). Indeed, the molecular mechanisms for tissue interaction, penetration, and remodeling that are seen during EMT in melanoma appear to have much in common with those seen with their ancestral cells undergoing similar processes in the neural crest. For instance, over 50 percent of genes associated with EMT and cell migration were induced in melanoma cells following exposure to the neural crest microenvironment (21, 22). Furthermore, transplanted melanoma cells respond to cues within the host embryonic microenvironment and subsequently mimic many aspects of neural crest cell motility without forming tumors (11, 22).

The key advantages of using this model are, first, the easy access to the developing embryo to visualize *in vivo* tumor cell behavior and its ability to respond to microenvironmental cues (23). Second, to clarify which specific factors and signaling pathways in embryonic development also participate in maintenance of tumor cell plasticity. Third, the legal and ethical restrictions are limited with early developmental stages of the chick embryo before hatching. Fourth, the transplants are not rejected (13). Finally, the short time frame required from start of the experiment to readout, it’s relative affordability and the lack of need for a specialized housing facility renders the chick embryo a suitable model system.

We and others have used this model to investigate the role of candidate genes in invasion *in vivo* by perturbing gene expression with morpholino or siRNA approach (10–12, 24). The ease of integrating this model with intravital imaging techniques and laser capture microdissection assisted gene profiling strategy has enabled the examination of dynamic temporal and spatial gene regulation exhibited by motile cells *in vivo* (22). Alternatively, invasive behavior of other tumor cells that share ancestral relationship with neural crest cells could be studied using this method (25). Although the avian embryo offers many advantages, it may be preferable to compare the results obtained in mammalian embryos, which are presumably more closely related to the environment found in human embryos (26).

We have previously reported the classification of metastatic human melanoma cell lines into epithelial- and mesenchymal-like cells based on gene expression profiling and functional assays (12). Herein, we have compared the behavior of epithelial- and mesenchymal-like melanoma cells *in vivo*. LM-MEL-8 is an epithelial-like cell line that lacks invasive ability *in vitro*, whereas LM-MEL-3 is a mesenchymal-like melanoma cell line with high invasive ability *in vitro*. Both cells were chosen for further study following transplantation into the chick neural tube where invasive ability was assessed *in vivo*. Although previous studies have transplanted melanoma cells at the head level of chick neural tube, we primarily performed transplantation at the trunk level of the chick embryos as the melanocyte progenitors arise predominantly from neural crest cells at this location.

## Materials

### Reagents

- Melanoma cell lines LM-MEL-3 and LM-MEL-8 were established from resected melanoma metastases. All tissue donors provided written informed consent for tissue collection and research, which was covered by protocols approved by the Austin Health Human Research Ethics Committee, Melbourne, Australia (approval number H2012/04446). All cell lines were matched with their donors by HLA-typing. Cells were cultured in RF-10 media (RPMI1640 supplemented with 10% fetal calf serum) as described previously (27).
- Adult normal human melanocytes (Lonza Australia).
- 1× Dulbecco’s phosphate buffered saline (DPBS) without calcium and magnesium (Life Technologies).
- TrypLE^TM^ (Life Technologies).
- Vybrant labeling DiO dye (Life Technologies).
- Fertilized chicken embryos (Research Poultry Farm, Australia).
- 70% ethanol.
- Sterile phosphate buffered saline and 1% penicillin/streptomycin (PBS-Pen/Strep).
- India ink (Pelikan Fount).
- Paraformaldehyde, 4% (wt/vol): add 30 ml of 1× DPBS to 10 ml of 16% (wt/vol) paraformaldehye (ProSciTech).
- 30% sucrose solution in DPBS.
- Tissue-Tek OCT solution (Olympus).
- Liquid nitrogen.
- Fluorescent mounting media (Dako).

### Equipment

- Egg incubator (Bell South).
- Dissection microscope.
- Fluorescent stereomicroscope with epi-illuminaton (SteREO Lumar V12 Carl Zeiss).
- Forceps size 3 and size 5.
- Scissors.
- Microscissors.
- Syringe (5 cc with a 18G needle and 1 cc with a 27G needle).
- Thin-walled glass capillaries (Harvard apparatus GC150T) pulled to generate a fine tip.
- Sharpened tungsten wire needles.
- Transparent tape.
- Sylgard petridish.

## Methods

**Figure 1:**
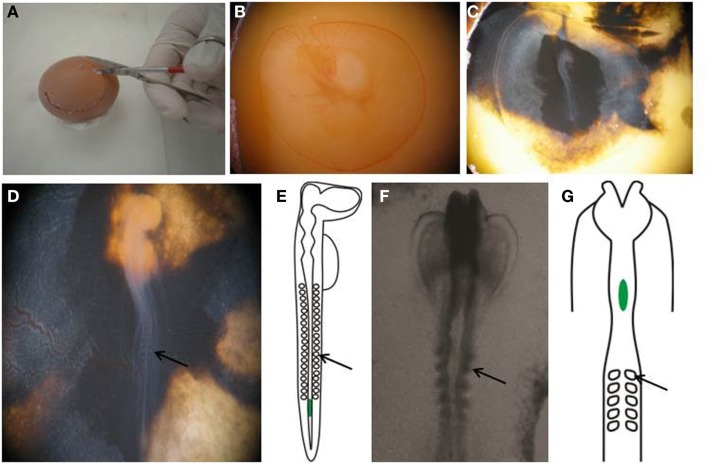
**Preparing egg for transplantation**. **(A,B)** Create a window in the eggshell and **(C)** inject with India ink to better visualize the embryo. **(D)** Perform staging of the embryo by counting the number of somites. **(E)** An E2.5, or HH stage 14 embryo, showing the location of the transplanted melanoma cells in green. **(F)** Wholemount view of the cranial region of an E1.5, or HH stage 8+ embryo. **(G)** Schematic of an E1.5/HH8+ embryo, showing the location of transplanted melanoma cells in green. Arrows point to somites.

**Figure 2:**
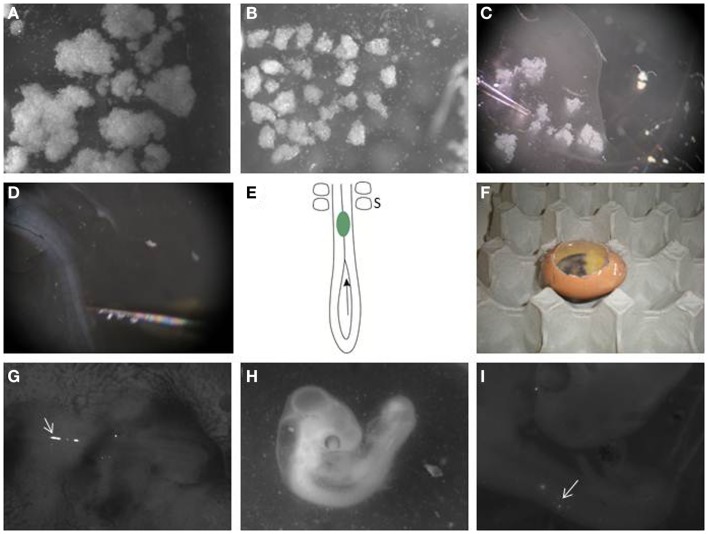
**Transplanting melanoma cells into the embryonic chick neural tube**. **(A)** Melanoma cells are labeled with DiO and allowed to grow as hanging drop culture. They form variable sized aggregates after 24 h. **(B)** Large clumps are dissected into similar sized small clusters using dissection tools. **(C)** A pulled glass pipette is used to aspirate a small cluster. **(D)** Melanoma cells are transplanted to the neural tube in the trunk region of the embryo. **(E)** Schematic of the caudal trunk region of an E2.5 embryo. The arrow is located in the posterior neuropore, showing the entry for a cluster of melanoma cells (green), which can then be pushed or injected into the neural tube so it lies just caudal to the most caudal somites (S). **(F)** Eggs are then re-sealed with transparent tape and allowed to incubate for 48 h. **(G)** Clusters of DiO-positive melanoma cells can be seen in the trunk neural tube immediately after injection when imaged using a fluorescence stereomicroscope (arrow). The head of the embryo is to the right. **(H)** Embryo is processed 2 days following injection. **(I)** DiO-positive melanoma cells can be seen under a fluorescence stereomicroscope in the trunk neural tube and in the surrounding tissue (arrow).

The experimental design of the protocol is depicted in Figures 1 and 2.

Incubation of fertilized eggs:

1. Place fertilized chicken eggs at 38°C in a humidified incubator for 48 h to allow the embryos to reach the desired stage of development (28).
2. Turn the eggs after 24 h to ensure that the yolk does not attach to the eggshell.

Labeling and hanging drop culture of melanoma cells:

3. Cells can be pre-treated with siRNA or other factors prior to culturing as a hanging drop (11–13).
4. Aspirate media and wash cell monolayer with 1× DPBS. Add 3 ml of TrypLE^TM^ to the flask and incubate at 37°C until cells detach.
5. Add 3 ml of media to the flask and transfer cells into a 15 ml conical tube. Centrifuge at 1800 rpm for 5 min.
6. Aspirate the media and suspend cells at a density of 1 × 10^6^ in 1 ml 1× DPBS.
7. Add 1 μl of the Vybrant cell labeling DiO dye to the cell suspension and mix well by pipetting.
8. Incubate cells with labeling solution at 37°C for 20 minutes. The DiO label is a lipophilic tracer dye. After 20 min, add 10 ml of RF-10 media.
9. Centrifuge the tubes at 1800 rpm for 5 min. Aspirate the supernatant and resuspend cells in media. Repeat the wash procedure two more times.
10. Resuspend cells in media at a density of 5 × 10^4^ cells per 25 μl media. Pipette four 25 μl drops of resuspended cells on the lid of a 35 mm culture petridish. Place the lid on the culture dish filled with 2 ml media. Incubate cells at 37°C for 24 h under standard cell culture conditions.

Preparation of eggs:

11. Rinse eggs with 70% ethanol and wipe clean. Do not turn eggs over at this time, the embryo will be on the uppermost side.
12. Mount the egg on the stage of a dissection microscope. Using a 5 cc syringe fitted with an 18 gauge needle, pierce a small hole in the eggshell and withdraw 3 ml of albumin from the caudal region of the egg.
13. Cut a circular hole in the eggshell with scissors to create a window to enable visualization of the yolk (Figures 1A,B).
14. Using a 1 cc syringe fitted with a 27 gage needle, inject 10% India ink in PBS-Pen/Strep below the blastodisc to enable better visualization of the embryo (Figure 1C).
15. Count the somite pairs in the embryo to determine the embryonic stage (28). Stage HH 12-14 embryos are used for this procedure (Figure 1D).
16. Make a small nick with a needle in the vitelline membrane overlying the most caudal end of the neural tube. Other procedures use similar processes to prepare chick embryos for grafting experiments (29–31).

Transplantation of melanoma cells into the neural tube:

17. Melanoma cells cultured as hanging drops form clusters (Figure 2A). Disaggregate the large cell clusters with needles into similar sized smaller clumps of approximately 500 to 1200 cells (Figure 2B). We use this method as single cells injected into the neural tube often float out. The clumps can be variable in size but they are malleable. The size of clump that can be inserted into the neural tube is limited by the width of the neural tube; therefore, the width of the clump is typically reproducible following placement in the neural tube, with greater variability seen in the length of the clump along the neural tube.
18. Aspirate a small clump of melanoma cells into the pulled glass pipette (Figure 2C).
19. Transplant the melanoma cells into the lumen of the neural tube in the trunk region, just caudal to the most caudal somites (Figures 1E and 2E). The glass pipette can be guided into the neural tube through the open neuropore, and cells injected into the neural tube caudal to the last somite (Figure 2E). Alternatively, a clump of cells can be maneuvered into the neural tube using tungsten wire needles through the open neuropore or a slit cut carefully into the dorsal neural tube closer to the most caudal somite. This is a delicate process with variable success rate. It is useful to incubate approximately 2 dozen eggs to generate roughly 6–12 injected embryos.
20. For transplantation into the cranial region, eggs are incubated for 1.5 days prior to injection, and embryos used between stages HH 8–10. Melanoma cells are placed into the neural tube at the level of rhombomere 1–2 (HH 8) to rhombomere 4 (HH 10) (Figures 1F,G).
21. Seal the window in the eggshell with transparent tape and re-incubate eggs in the incubator at 38°C for 48 h to allow melanoma cells to invade the host tissue (Figure 2F). Following injection, fluorescently labeled cells can be seen in the lumen of the trunk neural tube (Figure 2G).

Harvesting chick embryo for image processing:

22. After 2 days, select the surviving embryos and harvest the embryos individually and place it in 1× DPBS in a petridish (Figure 2H).
23. Under the fluorescence stereomicroscope, dissect out the membranes covering the embryo using microscissors and microforceps.
24. Place embryos dorsal side up with needles on a Sylgard petridish. Locate the fluorescent labeled melanoma cells within the embryo using the upright stereomicroscope (Figure 2I).
25. Whole mount pictures are captured of the embryo containing fluorescently labeled melanoma cells.
26. Using microscissors dissect the region of interest and fix it in 4% paraformaldehyde for 1 h on a shaker.
27. Wash the tissue in 1× DPBS and embed the embryos in 30% sucrose in DPBS overnight at 4°C.
28. Transfer the tissue into a cryomold filled with Tissue-Tek OCT Compound. Using forceps, position the tissue so cross-sections can be cut and remove air bubbles.
29. Freeze in liquid nitrogen. Section tissues at 20 μm using a cryostat. Sections can be frozen and stored prior to mounting.
30. Mount slides in fluorescent mounting media or counterstain with antibodies to visualize proteins of interest. DiO does fade over time; therefore, it is advisable to examine and image sections shortly after processing and mounting. Melanoma cells can also be detected using anti-human antibodies, such as Abcam ab7856 mouse anti-HLA DR + DP + DQ (CR3/43), used at 1:200 dilution with a fluorescent secondary anti-mouse antibody.

## Results and Discussion

**Figure 3:**
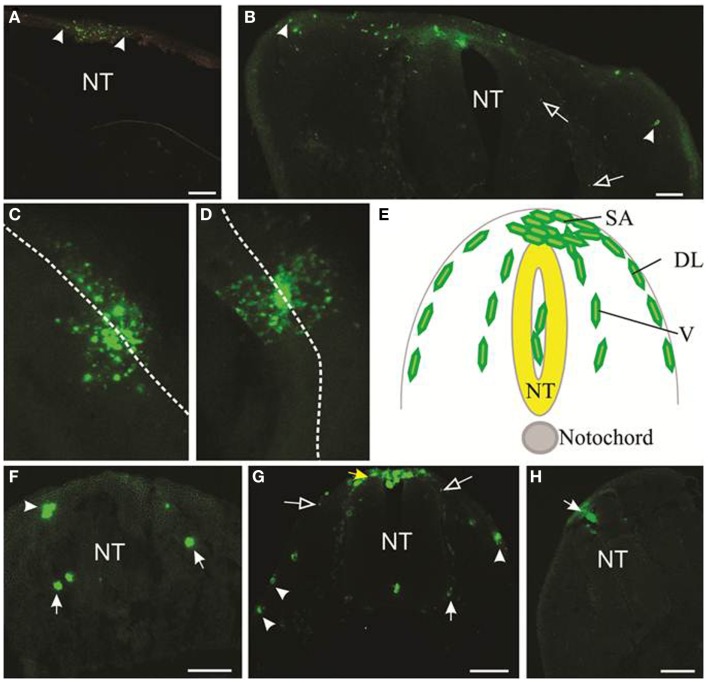
**Migration of melanoma cells from the neural tube**. Melanoma cells labeled with DiO are green. **(A,B)** Cross-sections of cranial **(A)** and trunk **(B)** levels showing the extent of migration of LM-MEL-3 cells at 4 days post-injection (DPI). Arrowheads indicate the cells that migrated the greatest distance from the middle of the dorsal neural tube. **(A)** LM-MEL-3 cells are located dorsal to the hindbrain. **(B)** LM-MEL-3 cells migrate further in trunk regions at 4 DPI. **(C,D)** Dorsal view of melanoma cells migrating from the neural tube at 2 DPI. White dotted line indicates the midline of the neural tube. **(C)** LM-MEL-3. **(D)** LM-MEL-8. **(E)** Schematic of a cross-section through the trunk region of the neural tube during neural crest or melanoma cell migration. Melanoma cells are injected into the lumen of the neural tube (NT) and some remain in this position without migrating. Cells emerge from the neural tube at the dorsal surface (top) into the staging area (SA). They then migrate along one of two major pathways: the dorsolateral pathway under the ectoderm (DL) or the ventral pathway (V). **(F,G)** Cross-sections of trunk at 2 DPI showing the location of **(F)** LM-MEL-3 and **(G)** LM-MEL-8 cells. Cells can be seen along dorsolateral (arrowheads) and ventral (arrows) pathways. The SA is indicated with a yellow arrow in **(G)**. **(H)** Melanocyte cells are found in the roof of the neural tube (arrow) but very few migrate outside the neural tube. NT, neural tube. Scale bar is 100 μm. Open arrows indicate speckles of DiO that have transferred to non-melanoma cells.

Typical results of motility of melanoma cells within the embryonic chicken are shown in Figure 3. DiO was used to mark the melanoma cells; however, anti-human HLA antibody also identifies melanoma cells (Figure S1 in Supplementary Material). We initially transplanted melanoma cells into both head and trunk regions of the neural tube. Analysis of two cell lines (LM-MEL-3, LM-MEL-8) showed that cells transplanted into the head region migrated far less readily than cells transplanted into the trunk region. A comparison of the migration of LM-MEL-3 at 4 days post-injection is shown for the cranial region (Figure 3A, *n* = 4) and the trunk (Figure 3B, *n* = 3). A similar difference in migration between head and trunk regions is also seen using LM-MEL-8 (*n* = 5 for each of head and trunk), which is a cell line with an epithelial-like phenotype and deemed to be non-invasive on the basis of *in vitro* characterization.

Previous work using the chicken embryo to assess melanoma migration has used cranial regions (11) and trunk (9). Kulesa et al. found that at cranial levels, a highly aggressive melanoma cell line C8161 migrated well, while a poorly aggressive cell line C8161 was less responsive (11). In our hands, both LM-MEL-3 (invasive mesenchymal-like) and LM-MEL-8 (non-invasive epithelial-like) cell lines showed poor migration at cranial levels and much greater migration at trunk levels, indicating that there were significant differences in the environmental signals between these regions. During normal development, the majority of melanocytes arises from trunk levels (18, 19), see Figure 3E. We concluded that the trunk was a preferable region for analyzing the migration of melanoma cells, but comparison of migratory ability between cells transplanted at cranial compared to trunk levels could be very informative.

During development, neural crest cells in the trunk migrate along two main pathways, the ventral pathway, which gives rise to neurons and glia in dorsal root and sympathetic ganglia, Schwann cells along nerves, melanocytes and adrenal chromaffin cells, and the dorsolateral pathway, which gives rise solely to melanocytes (19, 32–34). We analyzed cross-sections of embryos containing LM-MEL-3 and LM-MEL-8 cells to see if the cell lines followed these pathways. Cells from both LM-MEL-3 and LM-MEL-8 migrated from the trunk neural tube and could be seen outside the neural tube at 2 days post-injection by a dorsal view of whole mount embryos (Figures 3C,D). These migrated along the dorsolateral and ventral pathways (Figures 3F,G). We observed numerous cells located dorsal to the neural tube, a region termed the staging area, where neural crest cells are found prior to migration along a pathway (Figure 3E, yellow arrow, Figure 3G). Cells were also observed along the dorsolateral pathway adjacent to the ectoderm, along the ventral pathway in association with the dorsal root ganglia, and a small proportion of cells were observed in between the ventral and dorsolateral pathways. Neural crest cells are found at each of these locations (35). This finding is in line with earlier studies that have demonstrated that melanoma cells migrated along both dorsolateral and ventral routes (11, 13). Melanoma cells from another epithelial-like melanoma cell line LM-MEL-71 (Figure S2 in Supplementary Material) also showed migration outside the neural tube, along the dorsolateral and ventral pathways. EMT in melanoma often occurs with acquisition of motility and concomitant decrease in the expression of E-cadherin and gain in the expression of N-cadherin (12, 36). Culturing both epithelial- and mesenchymal-like melanoma cell lines as hanging drops do not induce changes in the expression of *E-cadherin* (epithelial marker) and *N-cadherin* (mesenchymal marker) (Figure S3 in Supplementary Material).

We also transplanted normal human melanocytes into the trunk region. A very small number of melanocytes migrated away from the neural tube, but this proportion was far less than the migration observed from melanoma transplantations. Some cells were observed in the roof plate of the neural tube, while others remained in the lumen of the neural tube (Figure 3H). Previous studies have reported that melanocyte aggregates integrated into the roof plate but did not migrate outside the neural tube (13).

The microenvironment of the neural tube of chick embryos affects the behavior of melanoma cells and promotes their migration along neural crest migratory pathways. In addition to chick embryos, zebrafish and mouse embryos have been used previously to provide either a receptive or potentially normalizing microenvironment that alters the behavior of human melanoma cells. Some factors active during gastrulation and early organogenesis within these embryonic microenvironments change the gene expression pattern of melanoma cells, limiting tumor formation, and enabling migratory potential (11, 26, 37). Bailey et al. identified a number of genes associated with EMT and migration upregulated in melanoma cells post exposure to the chick microenvironment (22). These gene products are excellent candidates to send and receive signals instructing and maintaining migratory potential in melanoma cells. Furthermore, epithelial-to-mesenchymal transitions are generally described as a process requiring external stimuli for their initiation (38). The process of neural crest development and the molecular mechanisms involved show similarity across species. For example, members of the BMP and Wnt families are involved in regulating neural crest induction and emigration in multiple species (39).

In conclusion, this model represents an accessible and potentially powerful system for examining the invasive behavior and remarkable plasticity of metastatic melanoma cells *in vivo*. It is proving helpful for identifying intrinsic and microenvironmental regulators and drivers of cellular migration. This should prove valuable for the identification and validation of molecules involved in metastatic behavior as well as for the development of therapies that target related pathways.

## Conflict of Interest Statement

The authors declare that the research was conducted in the absence of any commercial or financial relationships that could be construed as a potential conflict of interest.

## Supplementary Material

The Supplementary Material for this article can be found online at http://www.frontiersin.org/Journal/10.3389/fonc.2015.00036/abstract
