# Supplementary figures and images for: Embryonic Chicken Transplantation is a Promising Model for Studying the Invasive Behavior of Melanoma Cells
Source: Front Oncol. 2015 Feb 16;5:36. doi: 10.3389/fonc.2015.00036 (PMC4329807; doi:10.3389/fonc.2015.00036)

## Slide 1
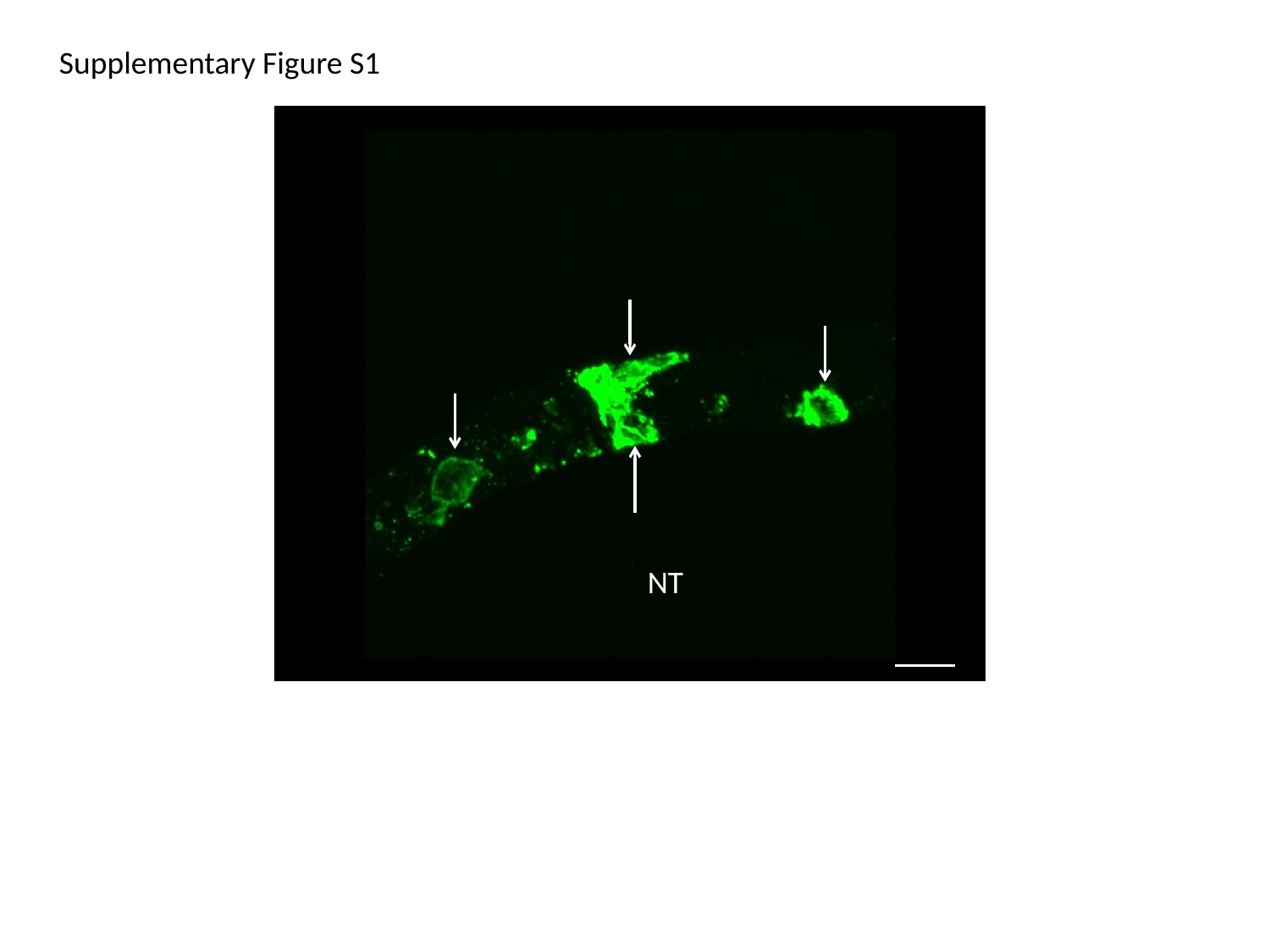

Supplementary Figure S1
NT

## Slide 2
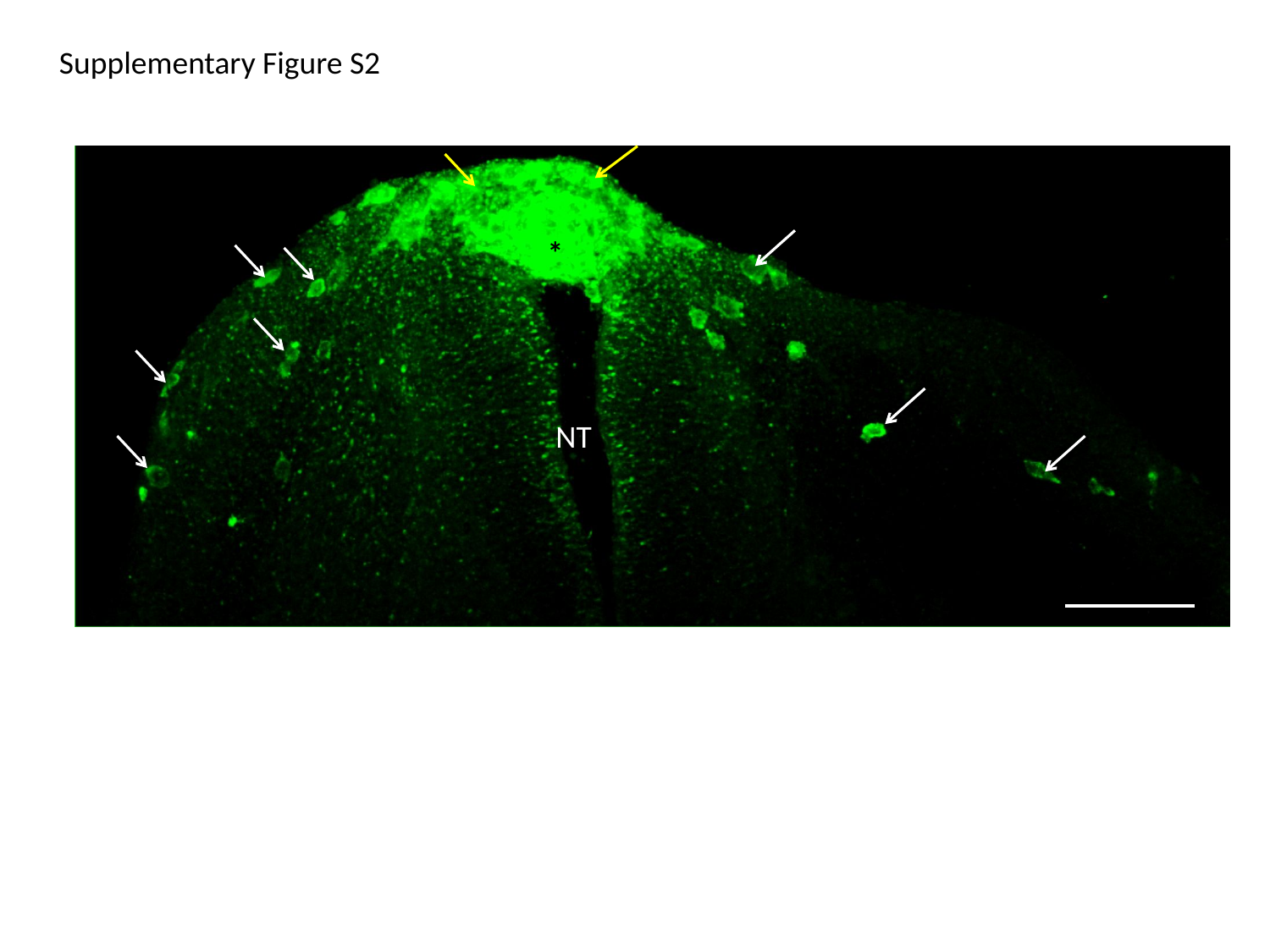

Supplementary Figure S2
*
NT

## Slide 3
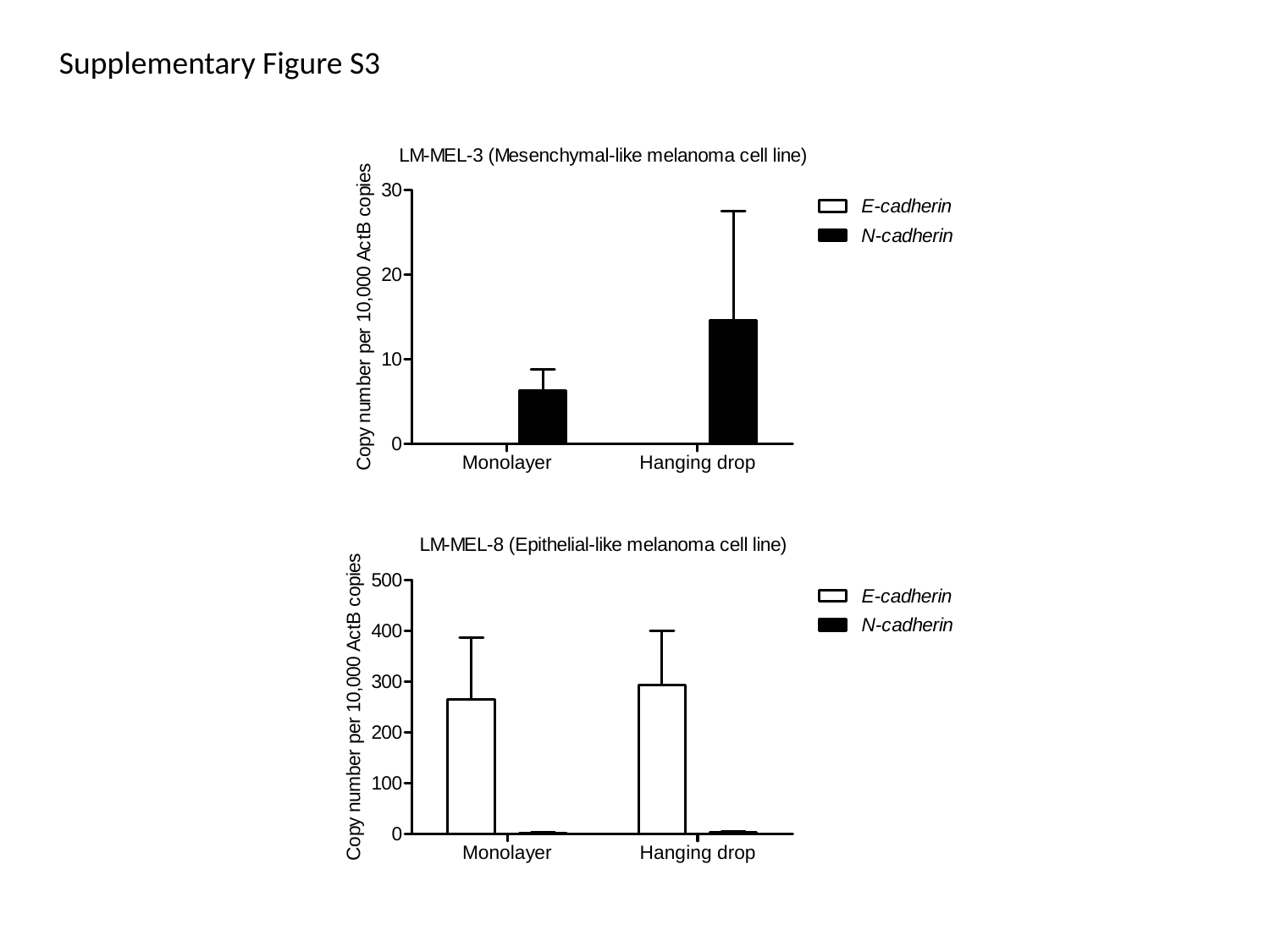

Supplementary Figure S3

Supplement: Supplementary file 1 [file Presentation_1.PPTX]
