# Supplementary material for: Embryonic Chicken Transplantation is a Promising Model for Studying the Invasive Behavior of Melanoma Cells
Source: Front Oncol. 2015 Feb 16;5:36. doi: 10.3389/fonc.2015.00036 (PMC4329807; doi:10.3389/fonc.2015.00036)
Supplement: Supplementary file 2 [file Data_Sheet_1.DOCX]

***Supplementary Material***

**Embryonic chicken transplantation is a promising model for studying the invasive behaviour of melanoma cells**

Aparna Jayachandran^1,2,3^, Sonja J. McKeown^4^, Briannyn L. Woods^4^, Prashanth Prithviraj^1,2^ and Jonathan Cebon^1,2,3^

^1^Cancer Immunobiology Laboratory, Ludwig Institute for Cancer Research, Melbourne-Austin Branch, Heidelberg, Victoria, Australia.

^2^Department of Medicine, University of Melbourne, Victoria, Australia

^3^School of Cancer Medicine, La Trobe University, Victoria, Australia

^4^Department of Anatomy and Neuroscience, University of Melbourne, Victoria, Australia

*Correspondence:

Jonathan Cebon

Cancer Immunobiology Laboratory

Ludwig Institute for Cancer Research

Melbourne-Austin Branch

Olivia Newton-John Cancer & Wellness Centre

Austin Health Level 5

Studley Road, Heidelberg

VIC 3084, Australia.

Phone: +61(3)9496-5462

[Jonathan.Cebon@onjcri.org.au](mailto:Jonathan.Cebon@onjcri.org.au)

1. **Supplementary data**

Quantitative real time PCR (qRT-PCR)

Total RNA was extracted using the RNEasy mini kit (Qiagen), and reverse transcribed using the High Capacity cDNA Reverse Transcription Kit (Applied Biosystems). Following reverse transcription, qRT-PCR was performed using the SensiFAST SYBR Lo-ROX kit (Bioline), and a Vii-A7 thermocycler (Applied Biosystems). The primers for *E-cadherin, N-cadherin* and *β-actin* (internal control) were synthesized by Sigma Aldrich and the sequences were as follows: *β-actin* (forward) 5'-CCA ACC GCG AGA AGA TGA-3' and (reverse) 5'-CCA GAG GCG TAC AGG GAT AG-3', *E-cadherin* (forward) 5’-gcc gag agc tac acg ttc a-3’ and (reverse) 5’-gac cgg tgc aat ctt caa a-3’, *N-cadherin* (forward) 5’-ctc cat gtg ccg gat agc-3’ and (reverse) 5’-cga ttt cac cag aag cct cta c -3’.

1. **Supplementary Figure Legends**

**Supplementary Figure S1: HLA staining.** Cross section of cranial region of chick transplanted with LM-MEL-3 melanoma cell line, labelled with anti-HLA antibody and anti-mouse 488 secondary antibody (Molecular Probes). NT indicates neural tube and arrows point to melanoma cells. Scale bar is 20μm.

**Supplementary Figure S2: Induction of motility in epithelial line upon injection into chick neural tube.** Cross section of trunk region of chick transplanted 2 days prior with LM-MEL-71, an epithelial-like melanoma cell line. Melanoma cells can be seen at the graft site (asterisk), and in both dorsal and ventral migratory pathways (arrows), and above the neural tube (NT) in the staging area (yellow arrows). Scale bar is 100μm.

**Supplementary Figure S3: Culturing melanoma cells as hanging drop does not induce an EMT.** qRT-PCR analysis revealed expression of E- and N-cadherins in (A) mesenchymal-like melanoma cell line LM-MEL-3 and (B) epithelial-like melanoma cell line LM-MEL-8 after culturing as monolayer or hanging drop.
